# The SABER School Feeding policy tool: a 10-year analysis of its use by countries in developing policies for their national school meals programs

**DOI:** 10.3389/fpubh.2024.1337600

**Published:** 2024-07-24

**Authors:** Linda Schultz, Alice Renaud, Donald A. P. Bundy, Fatoumata B. M. Barry, Luis Benveniste, Carmen Burbano de Lara, Mouhamadou Moustapha Lo, Jutta Neitzel, Niamh O’Grady, Lesley Drake

**Affiliations:** ^1^Research Consortium for School Health and Nutrition, London School of Hygiene & Tropical Medicine, London, United Kingdom; ^2^World Bank Group, Washington, DC, United States; ^3^World Food Programme, Rome, Italy; ^4^Partnership for Child Development, Imperial College London, London, United Kingdom

**Keywords:** SABER, school feeding, School Meals Coalition, human capital, education policy

## Abstract

Since its launch in 2011, 59 governments have used the World Bank’s Systems Approach for Better Education Results (SABER) policy tool to design their national school-based health and nutrition programs. This tool guides governments to self-evaluate their education system policies against international benchmarks and identify actionable priorities to strengthen national programs. Thirty-two of the 49 countries in sub-Saharan Africa (65%) have undertaken a SABER review, and globally the approach has been adopted by 68% of the world’s low-income countries and 54% of lower-middle-income countries. Analysis of 51 comparable SABER School Feeding surveys suggests that countries with longer established national school meals frameworks tend also to be more advanced in other policy areas, and vice versa. The SABER reviews consistently identify, perhaps predictably, that the weakest policy areas relate to program design, implementation and fiscal space. This analysis also found that the tool had an additional value in tracking the evolution of policies when implemented over several time points, and showed that policy areas become more advanced as national programs mature. These benefits of the tool are particularly relevant to the 98 countries that co-created the global School Meals Coalition in 2021. The Coalition member countries have the specific goal of enhancing coverage and support for the well-being of schoolchildren and adolescents affected by the school closures during the COVID-19 pandemic. The SABER tool has the demonstrated potential to implement, accelerate and track changes in school meals policy and, since it has been previously used by 74% (31/42) of low- and lower-middle-income countries in sub-Saharan Africa, is an already accepted element of the political economies of those countries and so has the potential to be deployed rapidly.

## 1 Introduction

In 2011, the World Bank developed an initiative intended to guide low- and lower-middle-income countries with designing, strengthening, and tracking their national education system policies. The Systems Approach for Better Education Results (SABER) initiative focused on various education domains, in recognition that a sound policy framework is the foundation for implementing policies effectively, monitoring implementation fidelity, and strengthening learning outcomes (1). Among these instruments were frameworks focused on the well-being of children, through better school health (SABER School Health) and the provision of school meals (SABER School Feeding).

The inclusion of multisectoral frameworks within the SABER initiative coincided with two milestones: first, with the recognition by the education sector of the important role of school health and nutrition interventions for the health, development and education of school children, and secondly, with country-led demand to expand national school meals programs as a social safety net during the 2008 Food, Fuel and Financial Crisis (2). No equivalent policy tool has emerged since the introduction of the SABER initiative; the tool has been widely used as part of the education sector planning process to develop a consensus view among multisectoral actors on the programmatic aspects of national school health and school feeding programs.

Health investments in children have tended to focus on the first 1,000 days of life, spanning conception to 2 years of age. Education investments largely cover the period from 5 years of age to the early twenties, a period increasingly designated as the ‘next 7,000 days’ (3). Investments in children through the next 7,000 days are essential to secure well-being into adult life and for the next generation (4), and similarly, educational achievement and human capital formation during this period depends on both good education and the well-being of school-age children (5). Enrolment in school, regular attendance and learning are often made more difficult by hunger or malnutrition (6, 7). Well-designed and effectively delivered school meals programs, especially when combined with complementary health and nutrition services, are among the most effective interventions available to governments seeking to transform education outcomes (4, 8). School meals are an effective strategy for promoting access to education: they typically led to a sustained increase of 9% participation, with higher rates in countries where girls’ participation in schooling has traditionally been low (9, 10).

The 2008 Crisis highlighted the interconnectedness of school-based health and nutrition interventions, as low-income countries leveraged World Bank emergency agricultural funds to expand the coverage of national school meals programs, with the multiple connected targets of improving social assistance, health, nutrition, education, and human capital (2). Although this was a response first seen in low-income countries, middle- and high-income countries similarly prioritized investments in school meals programs when faced with the subsequent global recession.

The following decade saw a steady growth of national school health and nutrition programs, with many delivering practical and affordable interventions at scale. By January 2020, school meals programs were delivered to more children in more countries than at any time in human history, reaching an estimated 388 million children daily (11). Ninety-three percent of governments implemented school meals in conjunction with complementary health and nutrition interventions; more than 100 countries offered school-based vaccination programs; nearly all countries integrated health education in their curriculum; and more than 450 million school-age children have been dewormed every year in schools in low- and middle-income countries (11–14). In India, for example, the National Deworming Day covers over 200 million children (15), and in tandem, the Government also implements allied programs, such as its Total Sanitation Campaign. Because the poorest and most marginalized children are often the students who have the most to gain from targeted education and health interventions (16), nearly three-quarters of national school health and nutrition programs include social safety nets as one of their objectives (17). Several diverse high-income and low-income countries provide free school meals to students from lower-income households, and in other settings, universal free school policies are expanding (18). Despite this progress, an estimated 73 million of the most vulnerable children had not yet been reached with daily school meals by 2020 (19).

The importance of these programs was again widely recognized in 2020 when schools worldwide were closed in response to the COVID-19 pandemic and governments experienced the counterfactual of trying to deliver health and nutrition interventions to school-age children in the absence of an education system (20). The closure of schools worldwide precipitated the largest education crisis in history, with more than 1.6 billion children deprived of schooling (21). From the perspective of the well-being of children, school closures abruptly ended a decade of global growth in school meals program, with around 370 million children suddenly deprived of their daily school meal at the height of the pandemic (22).

National political leaders formed the School Meals Coalition at the 2021 UN Food System Summit with the specific aim of rebuilding school-based services and protecting the human capital of schoolchildren. The School Meals Coalition, which is comprised of (now) 98 high-, middle-, and low-income countries and represents more than 63% of the world’s population, has three aims: (i) to restore access to school meals programs lost during the pandemic; (ii) help low-income countries reach the most vulnerable; and (iii) improve the quality, sustainability and scale of national school meals programs and complementary interventions (23). Now with additional energy and incentive, attention has once again turned to identifying appropriate policy tools to support national governments with strengthening the design of their national school meals programs to ensure that all children can benefit as countries build back from the pandemic.

This paper aims to document the uptake of the relevant SABER policy tools in low- and lower-middle-income countries over a 10-year period. This analysis is supported by an analysis of policy trends using comparable SABER School Feeding surveys, and in settings where the tool has been repeated, explores how the strengths of national policies have evolved over time. This analysis also aims to assess whether SABER would be an appropriate tool for countries to track their progression toward delivering more comprehensive and equitable school meals programs.

## 2 The history of the World Bank Systems Approach for Better Education Results framework

### 2.1 SABER: a policy tool to assess the quality of education policies across domains

One of the great successes of the Millennium Development Goals was achieving near universality in primary school enrolment. This achievement shifted the needle from a focus on equitable access to education to ensure the quality of education offered. It was during this period that the World Bank introduced its 10-year Education Sector Strategy, *Learning for All*, to advance learning and quality education in the low-resource settings. Notably, the strategy incorporated school meals and complementary school health interventions to improve learning readiness and encouraged the creation of a global knowledge base to guide country-level reforms of education systems (24).

**Figure 1:**
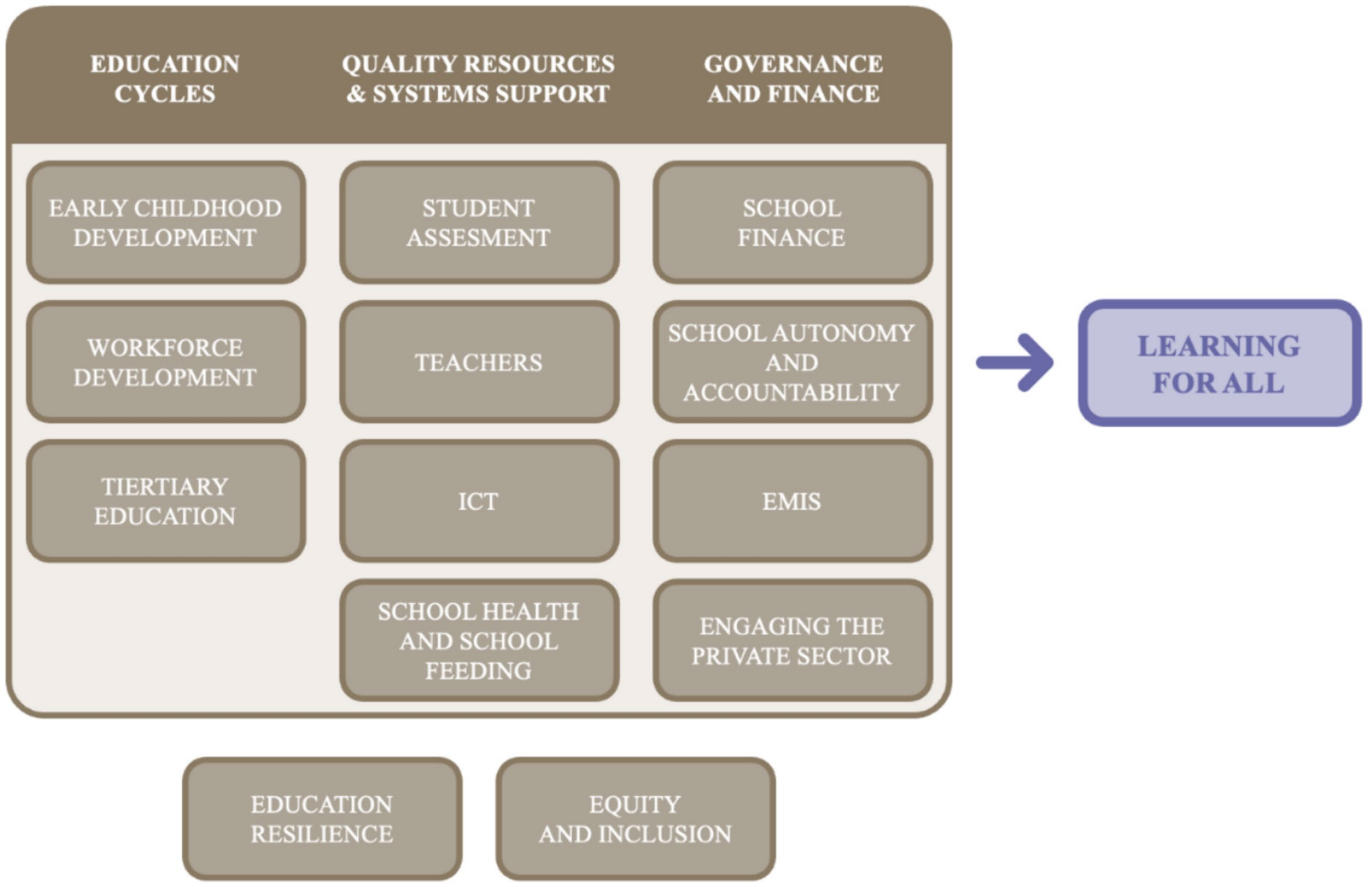
SABER policy domains. Source (1): ICT, Information and Communications Technology; EMIS, Education Management Information Systems.

The World Bank Human Development Network^1^ introduced the SABER initiative to help countries systematically collect information about the quality of their education policies for a wide range of education policy domains and identify actionable priorities for strengthening national education systems (Figure 1). Its emphasis on country-derived priority setting to strengthen the quality of education policies was in recognition that national policies can influence the whole of the results chain for learning, and that policies are sustained even following changes in political leadership.

To do this, SABER measures and benchmarks progress toward good practices for each policy area to inform a more in-depth analysis of policy and program implementation,^2^ using four policy indicator classifications:

1. *Latent:* Very little policy or programmatic implementation and/or development;
2. *Emerging:* Little policy or programmatic implementation and/or under development;
3. *Established:* Minimum policy or programmatic implementation; and
4. *Advanced:* Implementation of a comprehensive policy framework or suite of services.

In response to the growing awareness of the potential for school health and nutrition interventions to support readiness to learn and human capital formation during the next 7,000 days of life (5, 25), the World Bank convened a cross-sectoral advisory committee with expertise spanning all aspects of school health and nutrition^3^ to develop a specific policy SABER framework for school-based health and nutrition programs.

Recognizing that the cost and scale of school meals programs are significantly larger than other components of a comprehensive school health program, a separate framework on this topic was developed in collaboration with the UN World Food Programme (WFP). The SABER School Feeding policy tool is organized around five policy goals, in recognition that effective school meals programs have a national policy framework, stable and predictable funding, sufficient institutional capacity for implementation and coordination, sound design and implementation, and community participation (2, 26). The SABER School Feeding manual, questionnaire, scoring rubrics, framework rubrics, and report template were finalized through an iterative process with 30 countries in two sub-regions of Africa (27, 28).

### 2.2 The rapid and sustained uptake of the SABER School Feeding policy tool

Two conclusions from the 2009 World Bank *Rethinking School Feeding* analysis were that school meals offer benefits for the education sector while also serving as social safety nets, and that country capacity to fund and manage these programs increases as they mature (2). Spurred by these findings, WFP, the largest humanitarian agency worldwide and the largest provider of school meals among development agencies, developed a new policy in 2013 to support governments transition to national ownership of their school meals program, and identified SABER as the appropriate policy tool to support that vision (29). This tool has also been adopted by civil society organizations, such as the Partnership for Child Development, as part of a larger package of technical assistance to governments on their school health and nutrition programs.

**Figure 2:**
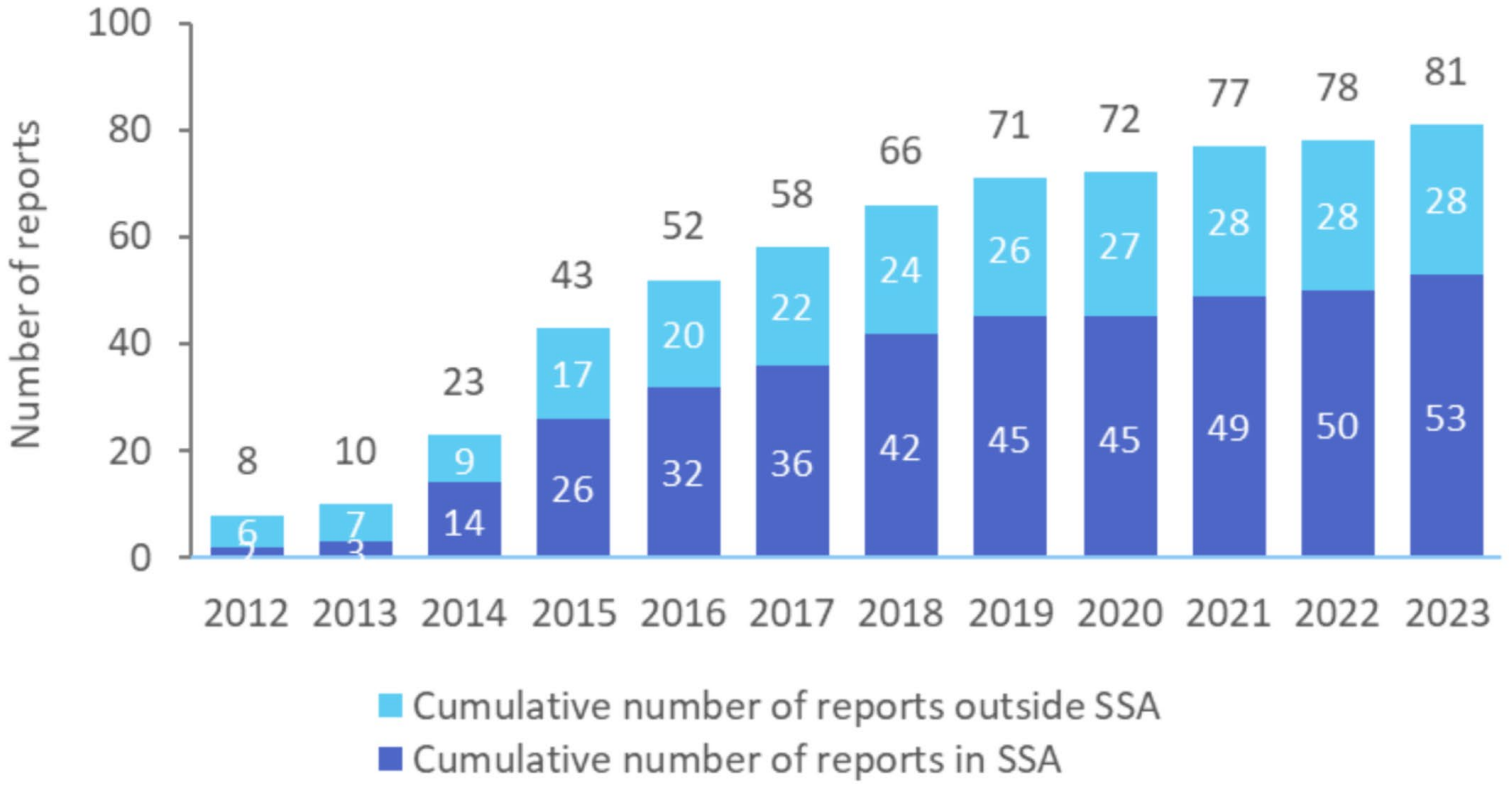
Cumulative number of SABER School Health and School Feeding exercises completed since 2012 globally and in sub-Saharan Africa (SSA), by year. The 81 reports include 67 SABER School Feeding exercises conducted in 52 countries, and 14 SABER School Health exercises in 14 countries. Twenty-two of these countries either repeated an exercise or conducted both the SABER School Health and School Feeding exercises.

Since 2012, countries have used the two SABER instruments to diagnose bottlenecks to sound school meals policy design and to co-create a strategy with diverse stakeholders to improve the implementation capacity of their national programs. The SABER School Health and SABER School Feeding policy tools have been conducted at least 81 times in 59 countries, with two-thirds of the instruments conducted in sub-Saharan Africa (Figure 2).

The majority of countries that have used the SABER tool have been in low- and lower-middle-income countries, which reflects that this tool was originally designed for uptake in World Bank client countries. At least 19 low- and 29 lower-middle-income countries have done either a SABER School Health or a SABER School Feeding exercise, which, respectively, represent 68% of low- and 54% of lower-middle income countries worldwide. Three quarters of all low- and lower-middle-income sub-Saharan African countries (74%) have conducted either a SABER School Health or a SABER School Feeding exercise (Table 1). These instruments, however, have been used by high- and upper-middle-income countries as well, suggesting that the tool remains useful in all settings.

**Table 1:** Uptake of SABER School Health and School Feeding surveys across low- and lower-middle-income countries (LICs and LMICs) and across sub-Saharan Africa (SSA) since 2012.

	Out of 28 LICs worldwide		Out of 54 LMICs worldwide		Out of 49 SSA countries		Out of the 24 SSA LICs		Out of 18 SSA LMICs	
	Number of LICs	Share of total LICs	Number of LMICs	Share of total LMICs	Number of SSA countries	Share of total SSA countries	Number of SSA LICs	Share of total SSA LICs	Number of SSA LMICs	Share of SSA LMICs
Countries that have completed either a SABER School Health or a School Feeding exercise (59 countries, 81 exercises total)	19	68%	29	54%	32	65%	18	75%	13	72%
Countries that have completed a SABER School Feeding exercise (52 countries, 67 exercises total)	19	68%	28	52%	32	65%	18	75%	13	72%
Countries in the analysis that have completed a SABER School Feeding exercise (45 countries, 51 exercises in total)	18	64%	24	44%	29	59%	17	71%	11	61%

Several externally designed surveys are available to provide year-on-year country comparisons of relevant school-based health and nutrition programs. The Global Survey of School Meal Programs (GSSMP), for example, was introduced in 2019. This survey supports the development of the WFP State of School Feeding bi-annual reports (11, 30). This questionnaire is shared with one or more appointed individuals in country to collect information on school feeding programs in country. To date, this questionnaire has been completed with 155 countries since it was introduced (31). The Health Behavior in School-Age Children (HBSC) Survey is a school-based survey that serves as a proxy for the quality of health service delivery in the school setting (12). This school-based survey has been administered in countries more than 50 times over a 40-year period, largely in North America and Europe (32), allowing for cross-country comparisons of school-based health programming over time.

No equivalent policy tool to SABER has emerged over this period. Its broad uptake in low- and lower-middle-income countries suggests that SABER has become an institutionalized policy instrument. Furthermore, the completion of the tool is demand-driven and engages all relevant actors across sectors to objectively surface policy and programmatic bottlenecks. The findings that emerge from this process supports governments with decision making around their own national program. It is because of this consensus approach that the tool is ill-suited for cross-country benchmarking.

## 3 The first global analysis of a decade of SABER School Feeding policy tool exercises

### 3.1 SABER School Feeding exercise collected from all World Bank regions

**Figure 3:**
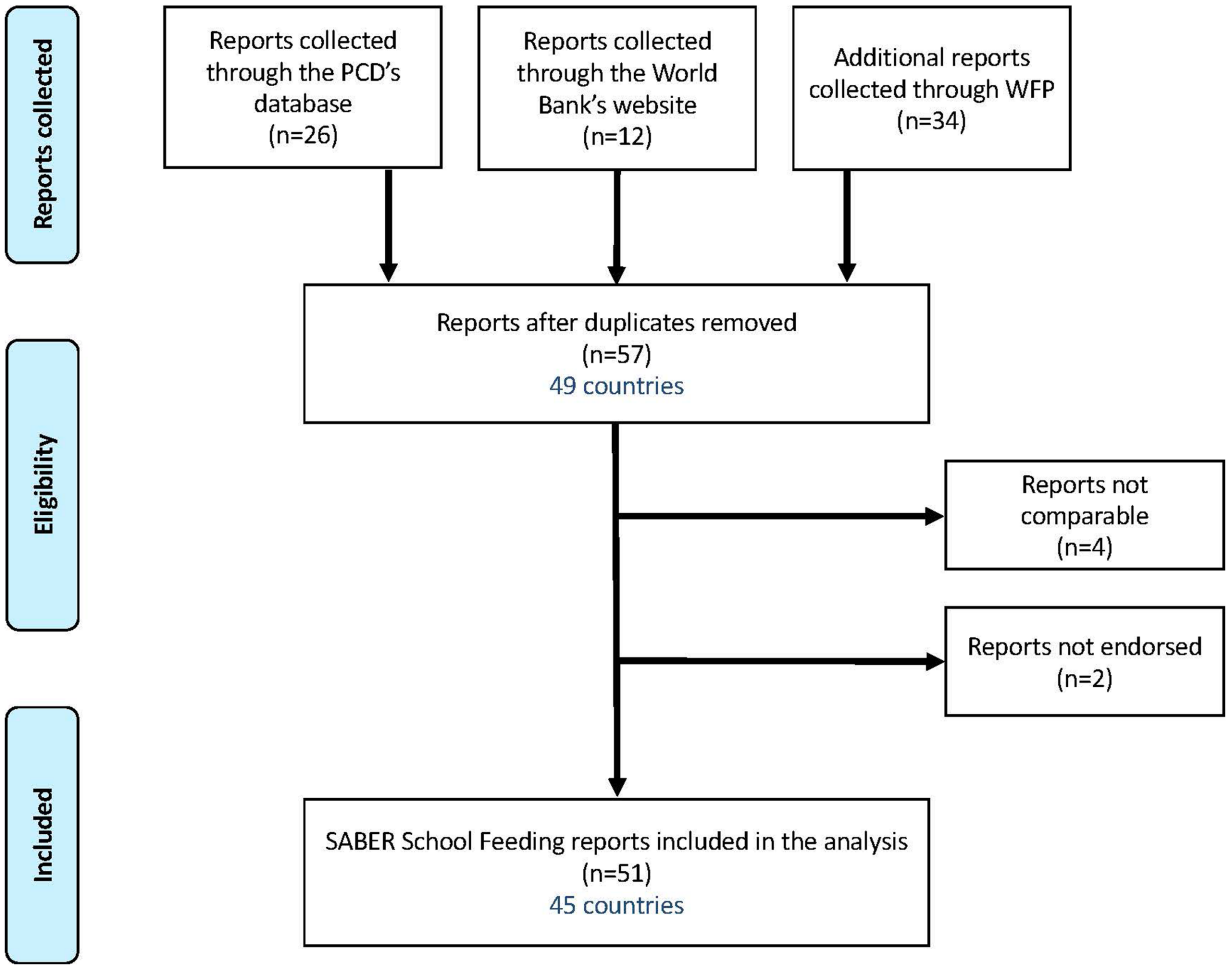
Flowchart of the search process to collect available and comparable SABER School Feeding reports for the analysis.

This is the first global analysis to assess how countries have utilized the SABER School Feeding policy tool over its decade of operation to guide national programs. To conduct this analysis, SABER School Feeding exercises were consolidated from three sources: (i) 34 reports were sourced from the WFP School-Based Programs Division; (ii) 26 SABER School Feeding reports were sourced from Partnership for Child Development; and (ii) 12 reports were sourced from the World Bank SABER website (33, 34). After removing duplicates, there were 57 SABER School Feeding reports, of which six were excluded due to incomplete data (4 exercises) and in response to country requests not to publish their data (2 exercises) (Figure 3). As a result, a total of 51 comparable SABER School Feeding reports were included in this analysis.^4^

The 51 reports were conducted between 2012 and 2021 and span 45 countries, with 6 countries conducting the assessment twice: Benin (2014, 2017), Ethiopia (2015, 2021), Kyrgyz Republic (2015, 2017), Tajikistan (2015, 2021), Togo (2012, 2016), and Tunisia (2014, 2021). An overview of the countries assessed in this study is presented in Table 2.

**Table 2:** Overview of SABER School Feeding exercises analyzed.

Country	Member of the School Meals Coalition, as of June 2024	World Bank region (Fiscal Year 2023)	World Bank country income category (Fiscal Year 2023)	Year(s) of SABER School Feeding exercise
Armenia	Yes	Europe and Central Asia	Upper-middle-income	2016
Benin	Yes	Western and Central Africa	Lower-middle-income	2014, 2017
Bhutan	Yes	South Asia	Lower-middle-income	2014
Bolivia	Yes	Latin America and the Caribbean	Lower-middle-income	2014
Burkina Faso	Yes	Western and Central Africa	Low-income	2015
Burundi	Yes	Eastern and Southern Africa	Low-income	2018
Cameroon	Yes	Western and Central Africa	Lower-middle-income	2016
Chad	Yes	Western and Central Africa	Low-income	201
Congo	Yes	Western and Central Africa	Lower-middle-income	2015
Côte d’Ivoire	Yes	Western and Central Africa	Lower-middle-income	2016
Democratic Republic of the Congo	Yes	Eastern and Southern Africa	Low-income	2018
Djibouti	No	Eastern and Southern Africa	Lower-middle-income	2017
Egypt	Yes	Middle East and North Africa	Lower-middle-income	2016
Ethiopia	Yes	Eastern and Southern Africa	Low-income	2015, 2021
Gambia	Yes	Western and Central Africa	Low-income	2014
Guinea	Yes	Western and Central Africa	Low-income	2018
Guinea-Bissau	Yes	Western and Central Africa	Low-income	2015
Haiti	Yes	Latin America and the Caribbean	Lower-middle-income	2015
Honduras	Yes	Latin America and the Caribbean	Lower-middle-income	2015
Indonesia	No	East Asia and Pacific	Lower-middle-income	2018
Jordan	No	Middle East and North Africa	Upper-middle-income	2016
Kenya	Yes	Eastern and Southern Africa	Lower-middle-income	2015
Kyrgyz Republic	No	Europe and Central Asia	Lower-middle-income	2015, 2017
Liberia	Yes	Western and Central Africa	Low-income	2019
Madagascar	Yes	Eastern and Southern Africa	Low-income	2014
Mali	Yes	Western and Central Africa	Low-income	2014
Mauritania	Yes	Western and Central Africa	Lower-middle-income	2015
Myanmar	No	East Asia the Pacific	Lower-middle-income	2017
Namibia	Yes	Eastern and Southern Africa	Upper-middle-income	2015
Nepal	Yes	South Asia	Lower-middle-income	2015
Niger	Yes	Western and Central Africa	Low-income	2017
Nigeria	Yes	Western and Central Africa	Lower-middle-income	2015
Philippines	Yes	East Asia and Pacific	Lower-middle-income	2019
São Tomé and Príncipe	Yes	Eastern and Southern Africa	Lower-middle-income	2016
Senegal	Yes	Western and Central Africa	Lower-middle-income	2014
Sierra Leone	Yes	Western and Central Africa	Low-income	2014
Sri Lanka	Yes	South Asia	Lower-middle-income	2015
Sudan	Yes	Eastern and Southern Africa	Low-income	2016
Tajikistan	Yes	Europe and Central Asia	Lower-middle-income	2015, 2021
Tanzania (Zanzibar)	Yes	Eastern and Southern Africa	Lower-middle-income	2015
Togo	Yes	Western and Central Africa	Low-income	2012, 2016
Tunisia	No	Middle East and North Africa	Lower-middle-income	2014, 2021
Uganda	Yes	Eastern and Southern Africa	Low-income	2014
Yemen	No	Middle East and North Africa	Low-income	2019
Zambia	Yes	Eastern and Southern Africa	Low-income	2016

**Figure 4:**
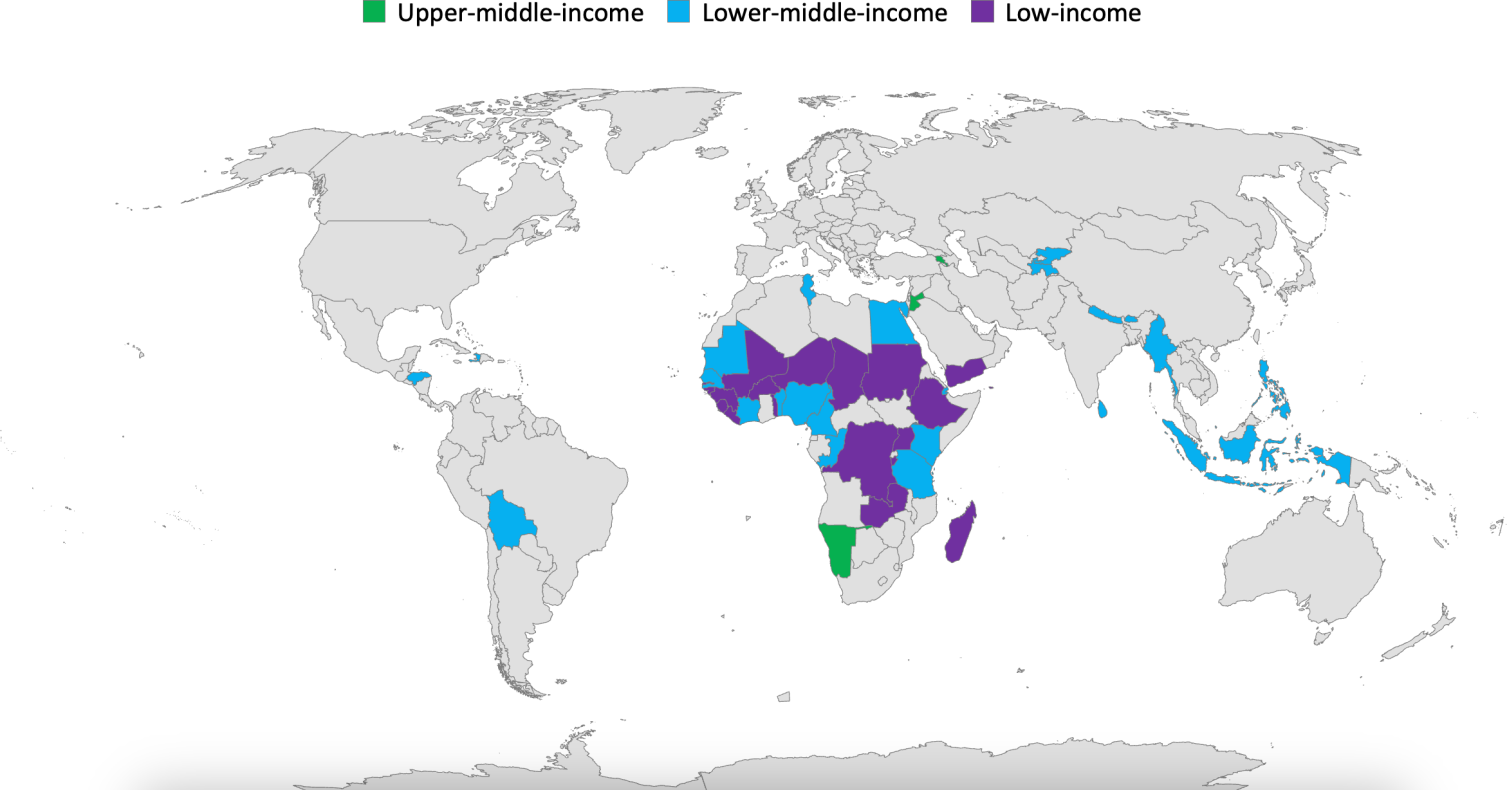
Geographical distribution and economic classification of the 45 countries that completed at least one SABER School Feeding exercise analyzed in this global analysis.

The 45 countries included in this analysis span all World Bank regions. Of the countries assessed, Western and Central Africa represents 37.7% (seventeen countries); Eastern and Southern Africa represents 26.6% (12 countries); Middle East and North Africa represents 8.9% (four countries); and the other regions (East Asia and the Pacific, Europe and Central Asia, Latin America and the Caribbean, and South Asia) each represents 6.7% (three countries in each region) (Figure 4).

### 3.2 Data analysis methodology

The development stages (Latent, Emerging, Established, or Advanced) for each Policy Goal and their supporting indicators were extracted and collated in a digital database. Patterns were analyzed by comparing the count of each development stage for each indicator and Policy Goal across all 51 exercises. For the countries that have repeated the SABER School Feeding instrument, an intra-country comparison of development stages for each indicator was conducted to show the evolution of school meals policies over the years. The five Policy Goals and related indicators of the SABER School Feeding policy tool are presented in Table 3.

**Table 3:** SABER School Feeding policy goals and indicators.

Policy Goal 1: Policy Frameworks	Indicator 1.1 National-level poverty reduction strategy or equivalent national strategy as well as sectoral policies and strategies (education sector plan, nutrition policy, social protection policy) identify school meals as an education and/or social protection intervention.
	Indicator 1.2 An evidence-based technical policy related to school meals outlines the objectives, rationale, scope, design, and funding and sustainability of the program and comprehensively addresses all four other policy goals.
Policy Goal 2: Financial Capacity	Indicator 2.1 National budget line(s) and funding are allocated to school meals; funds are disbursed to the implementation levels (national, district, and/or local) in a timely and effective manner.
Policy Goal 3: Institutional Capacity and Coordination	Indicator 3.1 Multisectoral steering committee coordinates implementation of a national school meals policy.
	Indicator 3.2 National school meals management unit and accountability structures are in place, coordinating with school-level structures.
	Indicator 3.3 School-level management and accountability structures are in place.
Policy Goal 4: Design and Implementation	Indicator 4.1 A functional monitoring and evaluation system is in place as part of the structure of the lead institution and used for implementation and feedback.
	Indicator 4.2 Program design identifies appropriate target groups and targeting criteria corresponding to the national school meals policy and the situation analysis.
	Indicator 4.3 Food basket corresponds to the objectives, local habits and tastes, availability of local food, food safety, and nutritional content requirements.
	Indicator 4.4 Procurement and logistics arrangements are based on procuring as locally as possible, taking into account the costs, capacities of implementing parties, production capacity in the country, quality of the food, and stability of the pipeline.
Policy Goal 5: Community roles	Indicator 5.1 Community participates in school meals program design, implementation, management, and evaluation and contributes resources (in-kind, cash, or as labor).

Simplified from (28).

When analyzing which Policy Goals were most mature, the Established and Advanced stages were consolidated and counted together. Similarly, the Latent and Emerging stages were consolidated to identify the weakest stages. For the research questions assessing the absence (NO) or presence (YES) of specific policy aspects, Latent and Emerging stages were considered as NO, and Established and Advanced stages were considered as YES, except when a more specific dichotomy was required. In those cases (i.e., indicators 4.3, 4.4, and 5.1), the Latent stage was considered as NO, and the Emerging, Established and Advanced stages were considered as YES.

### 3.3 Key findings

#### 3.3.1 Countries need the most support to strengthen the program design and implementation and the financial capacity of national school meals programs

The policy area with the most advanced development stages relates to Policy Goal 1, which considers whether school meals are included in a national poverty reduction strategy and whether a technical school meals policy exists. The analysis shows that 33% of low-income countries and 42% of lower-middle-income countries assessed scored either “Established” or “Advanced,” suggesting that over a third of the countries have a strong foundation for their national school meals programs.

**Figure 5:**
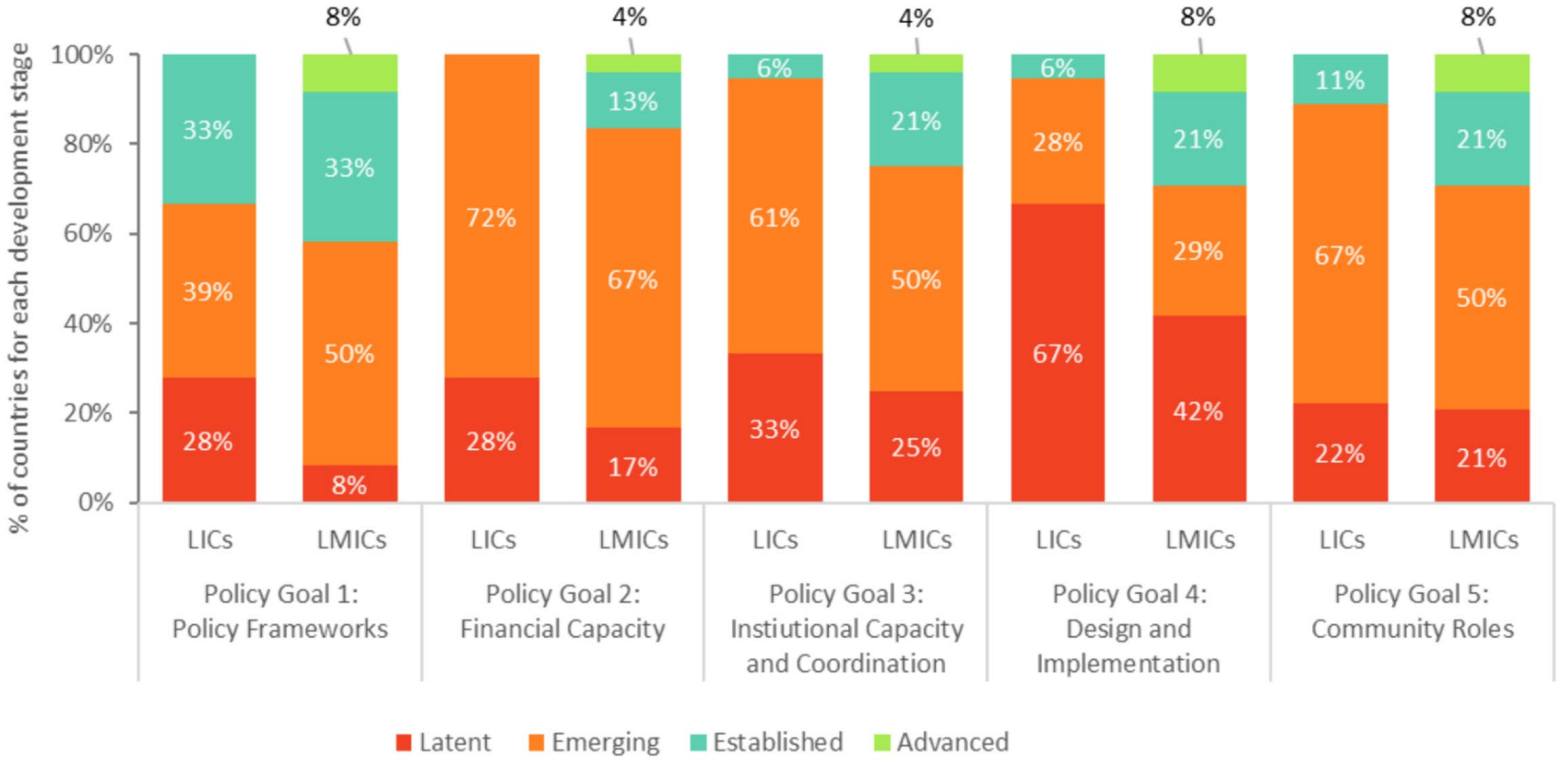
Comparison of the development stages of the SABER School Feeding Policy Goals between low-income and lower-middle-income countries.

On the other hand, program design and implementation (Policy Goal 4) is the most “Latent” development stages across the countries included in the global analysis (67% of low-income countries and 42% of lower-middle-income countries assessed). The policy area related to financial capacity is the weakest area, as 100% of low-income countries and 84% of lower-middle-income countries assessed score either “Latent” or “Emerging” for Policy Goal 2 (Figure 5).

In terms of program design and implementation, about a quarter of the low-income countries assessed have developed national standards for the food basket (28%) (indicator 4.3) and for procurement and logistics arrangements (28%) (indicator 4.4). Among the low-income countries assessed, none reported using monitoring and evaluation data to refine and update the standards periodically at the time of the assessment. On the other hand, half of the lower-middle-income countries assessed have developed national standards for the food basket arrangements (50%) and for procurement and logistics (58%). Among these countries, the Kyrgyz Republic (2017 exercise), Nigeria (2015 exercise) and the Philippines (2019 exercise) reported using monitoring and evaluation data to refine and update the standards periodically.

#### 3.3.2 Countries with mature policy frameworks for school meals are also more advanced in other policy areas

Among the countries that included school meals in a published national poverty reduction strategy or equivalent strategy, 39% also published a technical policy related to school meals. In comparison, only 6% of countries published a national school meals policy in the absence of a poverty reduction strategy.

**Figure 6:**
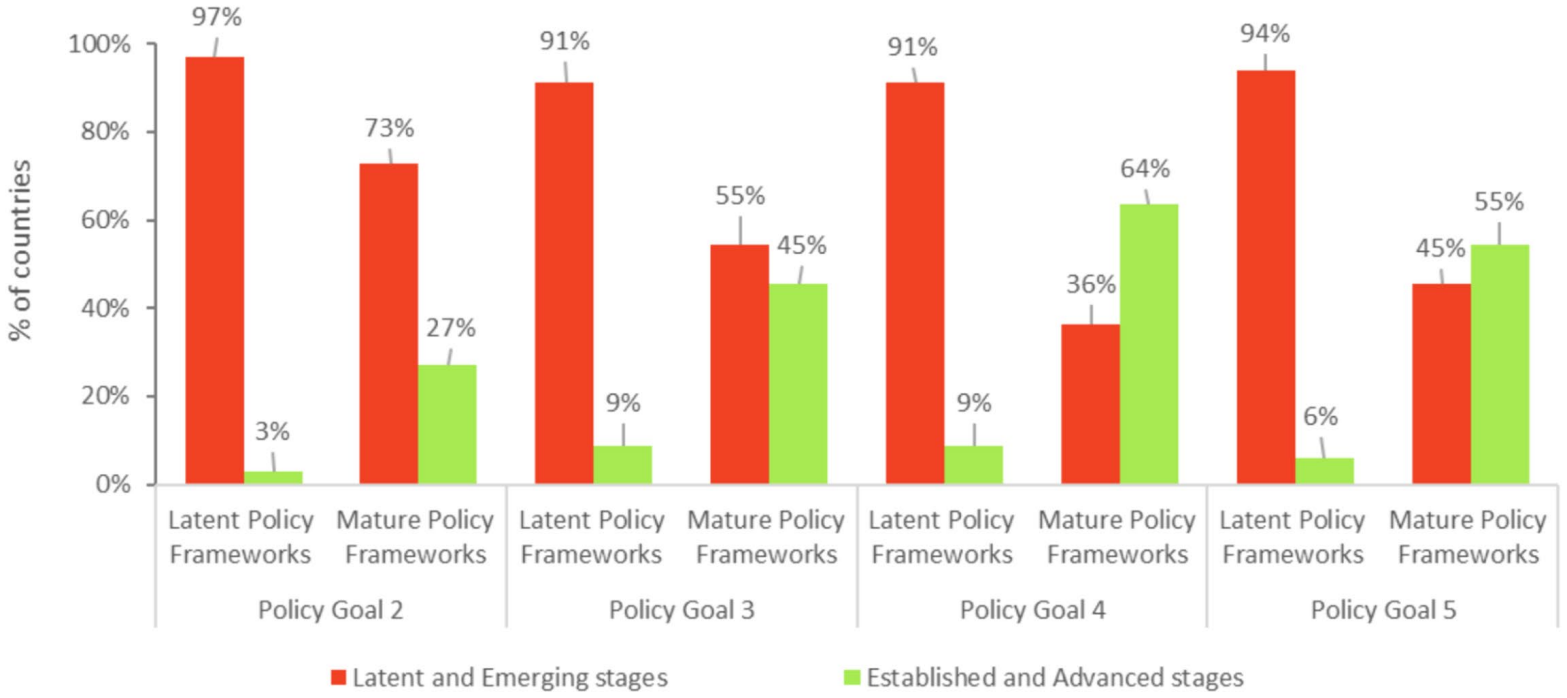
Comparison of development stages for Policy Goals 2, 3, 4 and 5 between countries with mature and latent policy frameworks (Policy Goal 1). In this graph, countries are determined to have a latent policy framework when Policy Goal 1 is assessed as “Latent” or “Emerging”; whereas mature policy frameworks are determined when Policy Goal 1 is assessed as “Established” or “Advanced”.

In addition, the analysis shows that countries with mature school meals policy frameworks (Policy Goal 1) tend to score more advanced stages for the other Policy Goals (Figure 6). For example, 64% of countries with a mature policy framework for school meals scored either “Established” or “Advanced” for the policy area related to program design and implementation (Policy Goal 4), against 8.8% of countries with more latent policy frameworks. Similarly, more than half (55%) of countries with a mature policy framework scored either “Established” or “Advanced” for the policy area related to community roles (Policy Goal 5), against 5.9% of countries with more latent policy frameworks.

#### 3.3.3 Lower-middle-income countries have more established multisectoral, ministerial-level steering committees to coordinate implementation of a national school meals policy than do low-income countries

**Figure 7:**
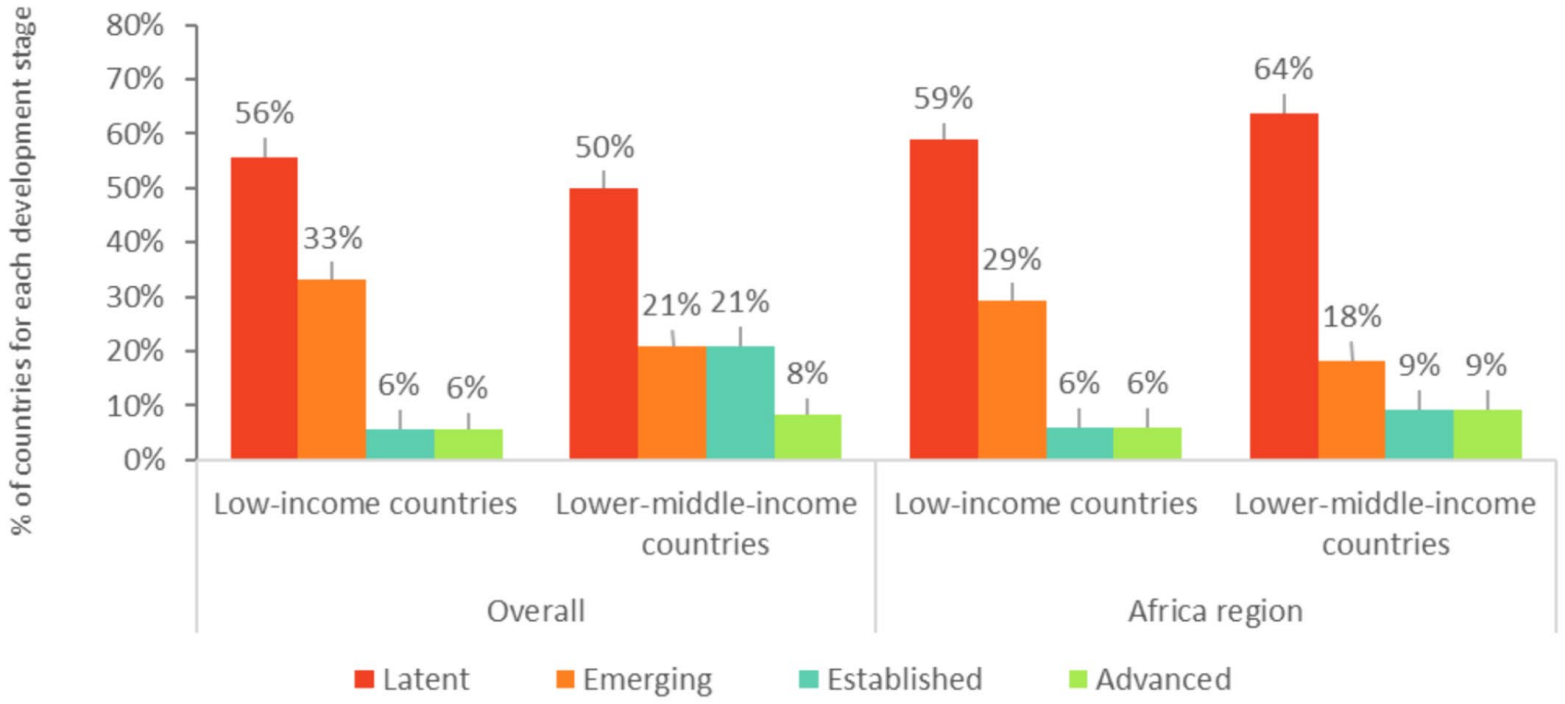
Development stages for a multisectoral steering committee coordination for school meals in low-income and lower-middle-income countries. Latent: absence of multisectoral steering committee coordination for school meals. Emerging, Established, and Advanced: existence of a multisectoral steering committee coordination for school meals. The World Bank Western and Central Africa, and Eastern and Southern Africa regions are analyzed together as the “Africa region”.

In most of the reports assessed, countries indicate that education is the leading sector responsible for the implementation of the school meals programs. Across regions and income categories, lower-middle-income countries tend to have more established multisectoral, ministerial-level steering committees than low-income countries to coordinate implementation of a national school meals policy. Overall, about a fifth of the countries assessed (22%) have a multisectoral school meals steering committee that coordinates the implementation of a national school meals policy (“Emerging” stage and above). Only a few of the low-income countries assessed (11%) have a multisectoral school meals steering committee that coordinates implementation of a national school meals policy (“Established” and “Advanced” stages), against nearly a third of the lower-middle-income countries assessed (29%). These trends are similar across the Africa region (Figure 7).

#### 3.3.4 School meals management committees are common, but community representation is rare

**Figure 8:**
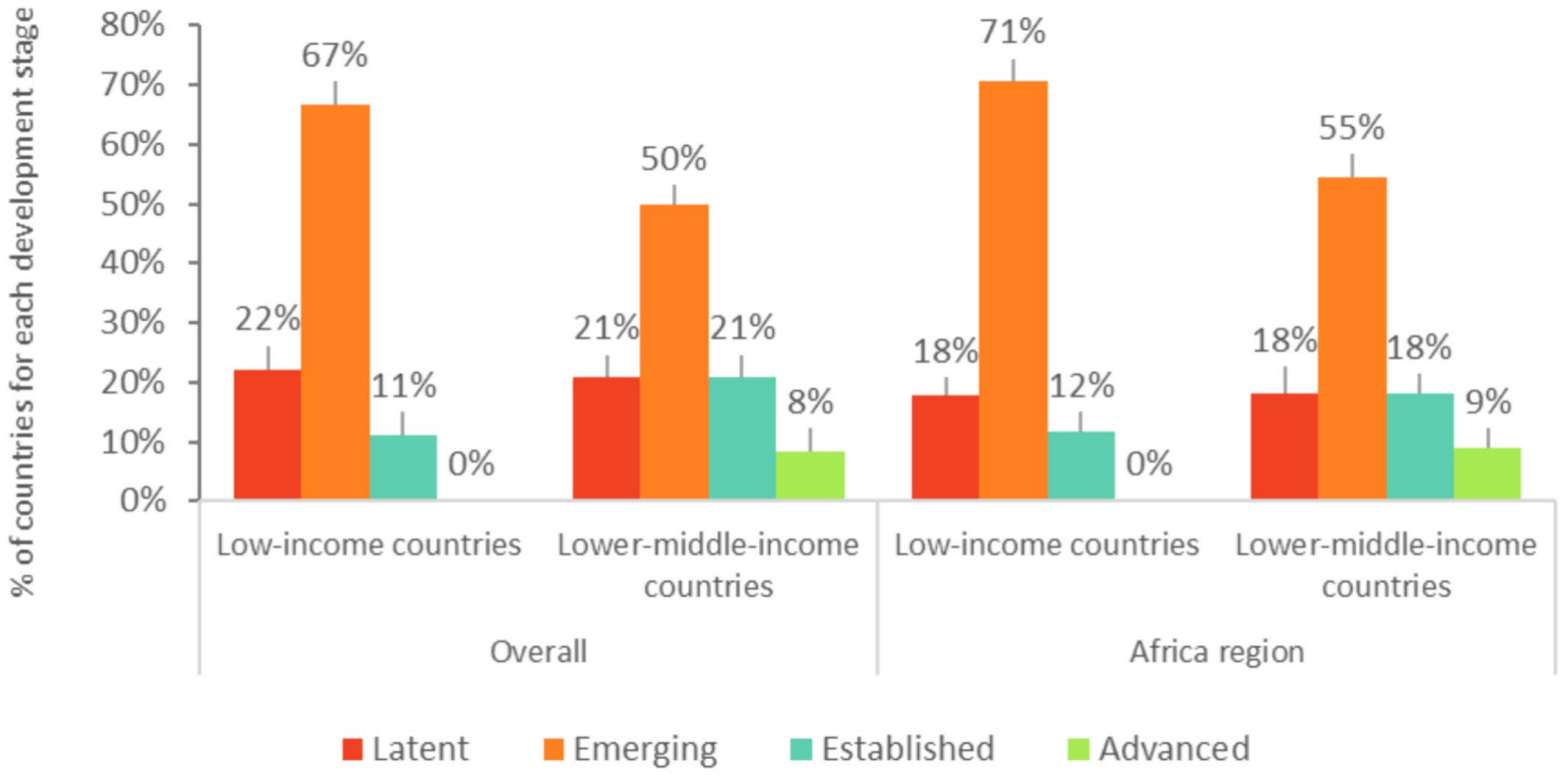
Development stages for community participation and accountability in low-income and lower-middle-income countries. Latent: absence of a system for community participation and accountability. Emerging, Established, and Advanced: existence of a system for community participation and accountability. The World Bank Western and Central Africa, and Eastern and Southern Africa regions are analyzed together as the “Africa region”.

Most of the countries assessed (78%) have a school meals management committee, but only 20% of the countries assessed report that the committee includes representatives of teachers, parents, and community members. Two countries report clearly defined responsibilities and periodic training at the time of the assessment (Kenya, 2015 exercise and the Philippines, 2019 exercise). A school meals management committee exists in most of the low-income (74%) and lower-middle-income countries (79%) assessed (Figure 8). Only two low-income countries (Niger, 2017 exercise and Ethiopia, 2021 exercise) report that the committee includes representatives of teachers, parents, and community members, but neither have clearly defined responsibilities and periodic training at the time of the assessment. In lower-middle-income countries, less than a third of countries assessed (29%) includes representatives of teachers, parents, and community members in their committees.

#### 3.3.5 Policy goals largely become more advanced as programs mature

**Figure 9:**
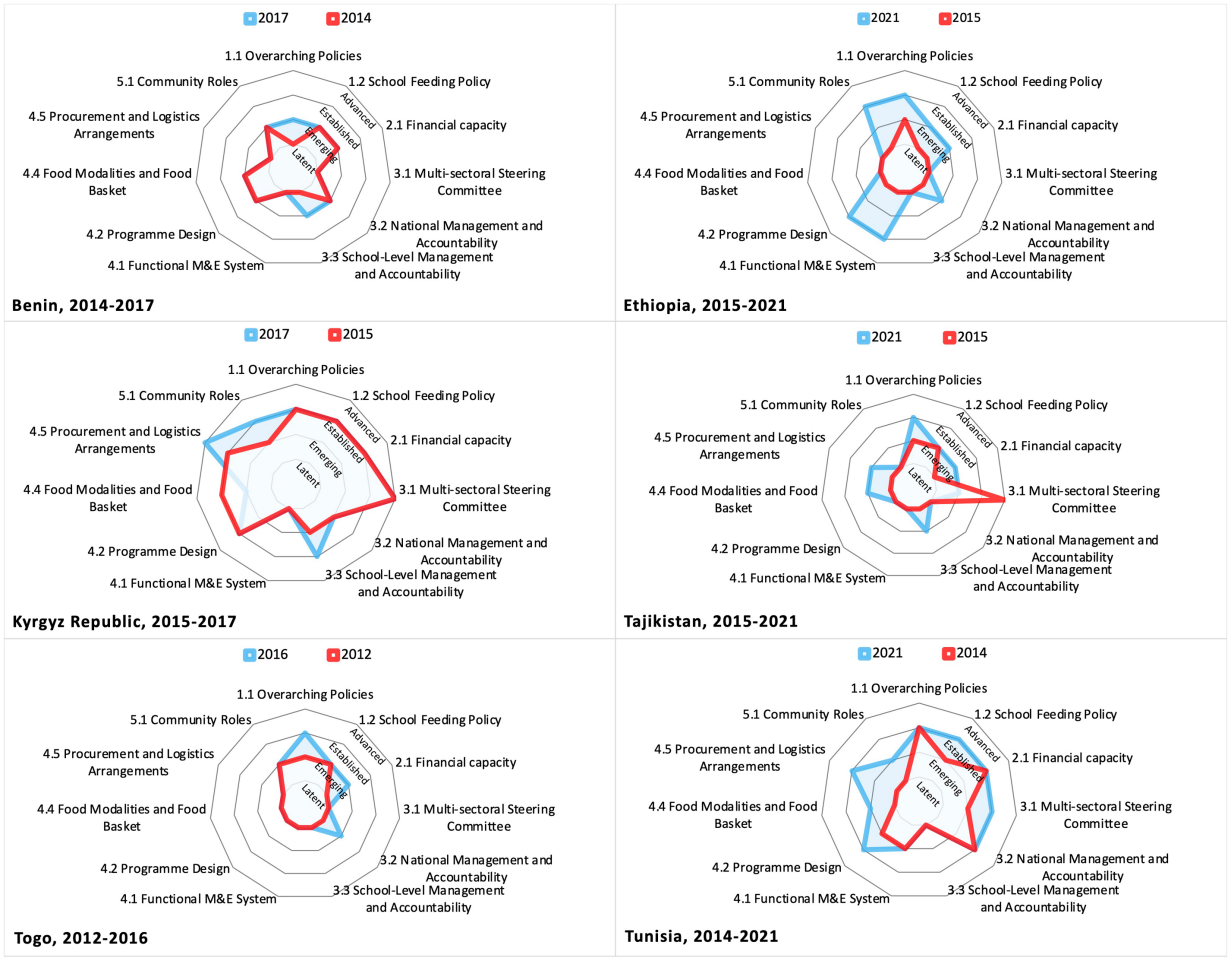
Evolution of school meals implementation capacity in countries that have completed multiple SABER School Feeding exercises. The red line and the blue line represent, respectively, the results of the first and second SABER School Feeding exercises. When only the red line is visible, it means that the result for a given indicator in the second exercise was unchanged from the first exercise.

Data from six countries that completed two comparable SABER School Feeding reports suggests that the capacity of countries to sustain school meals programs largely increases over time. In almost all policy domains, countries either improved their policy frameworks or held them constant (Figure 9). The analysis shows that development stages improved on 38% indicators, stayed the same on 59% indicators, and regressed on 3% indicators. The only instances of regression are for national standards on the food basket (indicator 4.4) were identified in the Kyrgyz Republic, which decreased from Established in 2015 to Emerging in 2017, and in Tajikistan for the establishment of a multisectoral steering committee (indicator 3.1), which decreased from Advanced in 2015 to Emerging in 2021.

The assessment of the data in the reports alone can suggest a correlation between the maturity of the school feeding policy frameworks and the evolution of policy level, but not a causal link. Indeed, the maturity of school meals national programs are affected by a number of external factors, such as economic shocks, disasters, and conflicts, as well as internal factors, such as the shifting baseline among policy makers, practitioners, beneficiaries of what is expected from a national school meals program. The influence of external and internal factors that affect the maturity of school meals national programs need to be further investigated.

## 4 Discussion

This global analysis identifies concrete opportunities to strengthen national school feeding policies and programs across the policy areas assessed. These findings suggest that program design, implementation, and the financial capacity are the weakest policy areas for school meals programs in low- and lower-middle-income countries. The sustainability of school meals policies requires not only political leadership and institutionalized frameworks, but also domestic funding for school meals and complementary services as part of a broader investment in the learner’s human capital. It may be particularly strategic to conduct SABER exercises before national poverty reduction or human capital strategies are due to be updated, as these national strategies were found to underpin the design of the technical policies related to school meals and complementary services.

An important finding from this analysis is that multisectoral coordination remains nascent in low-income settings, suggesting that particular attention to this area could strengthen the delivery of school meals programs in these settings. Effective policy implementation depends on the coordination of actors across different sectors, operating at the central-, sub-national, and school levels to procure and deliver large quantities of food to targeted schools, to ensure the quality of the food, and to manage resources efficiently and transparently. Efficient programs define the responsibilities of the multiple sectors involved, including from Education, Health, Nutrition, Finance, Agriculture and Social Protection, as well as sectors whose roles have only slowly become fully recognized, especially in the creation of human capital and the pursuance of goals related to gender equity and climate change (35). Having an agreed structure enables all sectors to contribute their experience, knowledge, and engagement to the procurement, delivery, and monitoring of the program (36).

It is important to note that the 51 exercises analyzed in this study were completed by 45 countries across a 10-year span (from 2012 to 2021), making cross-country comparison difficult. The reason for this challenge is multi-fold: principally, the original intent of the SABER instrument is not to rank national programs, but rather to support countries in identifying the specific elements of national policy frameworks that likely needed the most attention to improve system effectiveness and learning outcomes. Secondly, the surveys were conducted at varying time points across the past decade, eliminating the potential to provide a snapshot of national policies at a single point in time. Thirdly, the exercises reflect national policies at the time when they were completed. As shown by this analysis, countries score at more advanced stages across the Policy Goals as the school meals program matures, and national programs were at varying stages of maturity when the exercise was conducted.

For these reasons, there are limitations in drawing causal implications between the completion of a SABER policy tool and progress or regression in quality and sustainability of national programs over time. It would be beneficial to develop case studies to document how the results from the SABER exercise guided policy decisions and strengthened implementation fidelity over time. A similar analysis of the learnings that can be gleaned from the analysis of SABER School Health surveys conducted to date would similarly be worthwhile.

Among countries that have completed multiple surveys, there is an opportunity to explore how external (ex. natural disasters, economic shocks, conflicts, etc.) and internal factors (ex. shifting baselines in people’s expectations of the program) influenced how each successive SABER survey was completed. This effort would contextualize how weaker (or stronger) policy areas influence the other areas assessed. Recognizing the potential for additional countries to repeat the exercise over time, there may be value in including these questions in subsequent iterations of the survey.

## 5 Looking ahead: the potential for SABER to guide the School Meals Coalition with developing national commitments for school meals and tracking policy development

Global crises, including the COVID-19 pandemic, have led to a call for greater focus on national government ownership of programs designed to strengthen the human capital of a nation. National school feeding programs, for example, have seen a 4% decline in coverage as low-income countries between 2020 and 2022, despite global coverage rebounding by 7% from pre-pandemic levels (11).

A central role of the School Meals Coalition is to support governments with improving the quality and reach of their national programs. The SABER policy tool could serve a dual purpose toward this objective: Firstly, it could usefully guide the (now) 98 Coalition member states across country income categories with developing ambitious but realistic national commitments to improve and scale current programming. Commitments could include a focus on institutional arrangements for multisector coordination, innovation in program design and coverage, knowledge sharing, financing, among other categories.

Secondly, if administered routinely, countries would be able to use SABER to establish a baseline and track the development of their national school meals policy over time. Recognizing that 87% of the countries that have completed a SABER School Feeding survey to date are School Meals Coalition member states (45 countries), those countries would immediately be able to observe policy changes in their own context once the survey is repeated. Repeat surveys would show progresss toward the presence of a costed policy and budget line, national standards for school food, local procurement in school meals menus, and whether school meals are part of a complementary package of school health services.

SABER was always intended to be a long-term, regularly updated source of data and analysis, with indicators and benchmarks periodically revised to reflect advancements in the understanding of good practice for each domain and policy area (1). Given the complementarities between school meals and other school-based health interventions, the World Bank and WFP, have combined key elements of the SABER School Feeding and SABER School Health framework into a single, comprehensive policy tool. ‘Healthy-SABER’ is envisaged to further engage multisectoral actors in the design of effective and holistic school health policies and clarify key areas for further investment.

Looking ahead, the policy tool could be further expanded to include indicators for topics requiring increasing attention, such as planetary health and the food systems that underpin school meals programs. School meals represent more than 70% of all publicly managed food systems in many countries, making it possible for policy changes to national school meals programs to immediately strengthen its response to environment and climate concerns and, through the power of procurement, help change agricultural practices and shorten supply chains in the longer term (37).

An immediate global good would be to identify a permanent open-access platform for all reports and publish the backlog of existing SABER School Feeding to improve the availability of quality data in this area. A Coalition-led Data and Monitoring Initiative can serve as a repository for these reports, which contain useful supplementary data and indicators on programs across national programs. In the interim, the progress of the SABER tool by countries will be reported to the School Meals Coalition through the WFP biennial State of School Feeding reports.

## 6 Summary conclusions

The World Bank SABER policy tool helps countries systematically collect information about the quality of their school meals policies and identify actionable priorities. Since its introduction, 68% of the world’s low-income countries and 54% of lower-middle-income countries have used this tool to assess the strength of their national policies for school meals and complementary programs. SABER is unusual in that it is a government-led, government-completed process and, perhaps uniquely, engages stakeholders from all relevant sectors, including from health, education and agriculture, which helps to achieve a consensus view on the ambitious but realistic national commitments to strengthen current school meals programming. This approach helps ensure that policies are sustained even when there are changes in political leadership. Some countries found the policy tool useful enough to repeat the exercise, demonstrating how SABER can have a secondary purpose of establishing a national baseline against which countries can track their policy progression toward good practice. The adoption of school-based programs to support schoolchildren has gained momentum from the creation of the global, country-led School Meals Coalition in 2021 in response to pandemic-related school closures with the shared vision of improving the quality and reach of their national school meals programs. Nearly three-quarter of all low- and lower-middle-income countries across the Africa region have already conducted a SABER exercise, and repeating the exercise now would allow these countries to immediately observe the degree to which their policy changes have been effective in their own context.

Over the last decade the SABER tool has been adopted world-wide, particularly in low- and lower-middle-income countries and in Africa, and is today part of the political economy of nations and an institutionalized mechanism for governments to self-assess and strengthen their national school meals programmes. Going forward, SABER is likely to become an increasingly important tool for the member countries of the global School Meals Coalition.

## Author contributions

LS: Formal analysis, Writing – original draft. AR: Formal analysis, Writing – original draft. DB: Writing – review & editing. FB: Writing – review & editing. LB: Writing – review & editing. CB: Writing – review & editing. MM: Writing – review & editing. JN: Writing – review & editing. NO’G: Writing – review & editing. LD: Writing – review & editing.

## Footnotes

^1^The SABER initiative was developed by the World Bank Human Development Network, Education Department with leadership from Donald Bundy, Angela Demas, Robin Horn, Elizabeth King, Harry Patrinos, Halsey Rogers, among others. The Australia Department of Foreign Affairs and Trade and the United Kingdom Department for International Development (now FCDO) provided support for the creation of this initiative.

^2^The good practices outlined in the SABER frameworks were determined through literature reviews and practitioner input to identify policy and institutional choices most often seen in the best-performing and fastest-improving education systems, i.e., (i) a national policy framework, (ii) sufficient institutional capacity for implementation and coordination, (iii) stable funding, (iv) sound design and implementation, and (v) community participation.

^3^The Expert Advisory Committee included representatives from GlaxoSmithKline, International Food Policy Research Institute, London School of Hygiene and Tropical Medicine, Partnership for Child Development, Save the Children, UNICEF, the World Bank, WFP and WHO.

^4^Additional SABER School Feeding exercises have been conducted since 2021 but were excluded as the reports were not yet endorsed at the time this analysis was conducted.
